# Circadian Expression of TIMP3 Is Disrupted by UVB Irradiation and Recovered by Green Tea Extracts

**DOI:** 10.3390/ijms20040862

**Published:** 2019-02-16

**Authors:** Sunyoung Park, Eun-Soo Lee, Nok-Hyun Park, Kyeonghwan Hwang, Eun-Gyung Cho

**Affiliations:** Basic Research & Innovation Division, R&D Unit, AmorePacific Corporation, 1920 Yonggu-daero, Giheung-gu, Yongin-si, Gyeonggi-do 17074, Korea; sypark@amorepacific.com (S.P.); soopian@amorepacific.com (E.-S.L.); aquareve@amorepacific.com (N.-H.P.); khhwang@amorepacific.com (K.H.)

**Keywords:** circadian rhythm, TIMP3, AQP3, UVB, anti-inflammation, *Camellia sinensis* leaves

## Abstract

The human skin is the outermost physical barrier and has its own circadian machinery that works either cooperatively with the central clock, or autonomously. Circadian rhythms have been observed in many functions related to epidermal homeostasis including hydration and inflammation, and this functional oscillation is disturbed by ultraviolet radiation (UVR), which is a strong environmental cue. Among the genes estimated to show circadian expression in the skin, metalloproteinase inhibitor 3 (*TIMP3*), has a rhythmic expression in synchronized human keratinocytes similar to that of the core clock gene *PER1* and an epidermal circadian regulatory gene, aquaporin 3 (*AQP3*) but was antiphase to the core clock gene *BMAL1*. Tumor necrosis factor-α (TNF-α), the regulatory target of TIMP3 via a disintegrin and metalloproteinase domain 17 (ADAM17), was inversely regulated when TIMP3 expression was downregulated by ultraviolet B (UVB) treatment. When synthetic TIMP3 peptides were applied to the cells, the secretion of TNF-α did not increase following the UVB treatment. Similar to TIMP3 peptides, *Camellia sinensis* leaf-derived extracts showed a distinguishing efficacy in recovering TIMP3 expression, downregulated by UVB treatment. Together, our results suggest that TIMP3 reversely mediates UVR-induced inflammation by being highly expressed during the daytime; therefore, recovering the circadian expression of TIMP3 using synthetic TIMP3 peptides or bioactive natural ingredients could at least in part inhibit the UVR-induced cellular phenomena.

## 1. Introduction

Most organisms have a circadian clock system that regulates daily oscillations of physiological functions. When light enters the retina, the central clock located in the suprachiasmatic nucleus (SCN) synchronizes peripheral clocks located in tissues, such as the skin, through hormones and neuronal signals [1,2,3]. The molecular mechanism of circadian oscillation of clock genes has been previously well-described [1]. As the outmost physiological barrier of the organism, the skin performs functions that protect organisms from chemical, physical, and pathogenic environmental stressors, and are critical for maintaining homeostasis in the body. Skin functions such as transdermal water loss, stratum corneum hydration, skin surface pH and temperature regulation, and barrier recovery rate are known to show circadian rhythms [4,5,6]. In addition to skin tissue, cultured skin cells such as keratinocytes, melanocytes, and fibroblasts also display distinct circadian machineries [7]. Recently, molecular links between the circadian clock and skin hydration, and psoriasis-like inflammation have been shown to be mediated through aquaporin 3 (AQP3) in keratinocytes and IL-23R in γδ^+^ T cells, respectively [8,9]. AQP3 is expressed in the epidermis and suprabasal layers, and helps in the transport of water and glycerol, between and into cells. In the previous report, AQP3 exhibited significant oscillations in HaCaT cells that were regulated by the CLOCK/BMAL1 heterodimer, which suggested a molecular link between the circadian clock and skin hydration [8]. 

Among various stimuli in the environment, ultraviolet rays (UVRs) are the most well-known causative agent to induce mutations in DNA and to modify gene expression patterns in the skin. Upon exposure to ultraviolet B (UVB), UV-specific inflammation- and stress response-related transcripts are significantly upregulated, but metabolism- and adhesion-related transcripts are strongly downregulated in human keratinocytes [10]. In addition, core circadian clock genes are downregulated by UVB treatment, which can be explained by the altered expression of clock-controlled genes (CCGs) [11]. 

Among the genes estimated to show circadian expression in the skin, we previously identified metalloproteinase inhibitor 3 (*TIMP3*) as a novel CLOCK-dependent diurnal gene and demonstrated the importance of its circadian expression in UVR conditions [12]. In this study, we showed that *TIMP3* mRNA was oscillating periodically in synchronized human keratinocytes, similar to the core clock gene *PER1* and the epidermal circadian regulatory gene *AQP3*, but was in antiphase to the core clock gene *BMAL1*, suggesting that TIMP3 in the skin may be highly expressed during the daytime but lowly expressed during the night-time. The downregulated expression of TIMP3 upon UVB irradiation and the consequent increase in tumor necrosis factor-α (TNF-α) secretion was reversed by a treatment with synthetic TIMP3 peptides. We also found that green tea extracts could recover the disrupted *TIMP3* expression in UVB conditions. These results suggest that TIMP3 plays a role as a protector against UV-induced cellular responses during the daytime in the human skin. Therefore, synthetic TIMP3 peptides or green tea extracts could be used as cosmetic ingredients to improve TIMP3 expression during the daytime when TIMP3 expression may be downregulated by UVR.

## 2. Results and Discussion

### 2.1. The Expressions of AQP3 and TIMP3 Oscillate Periodically, Similar to That of PER1 but Are in Antiphase to BMAL1

**Figure 1:**
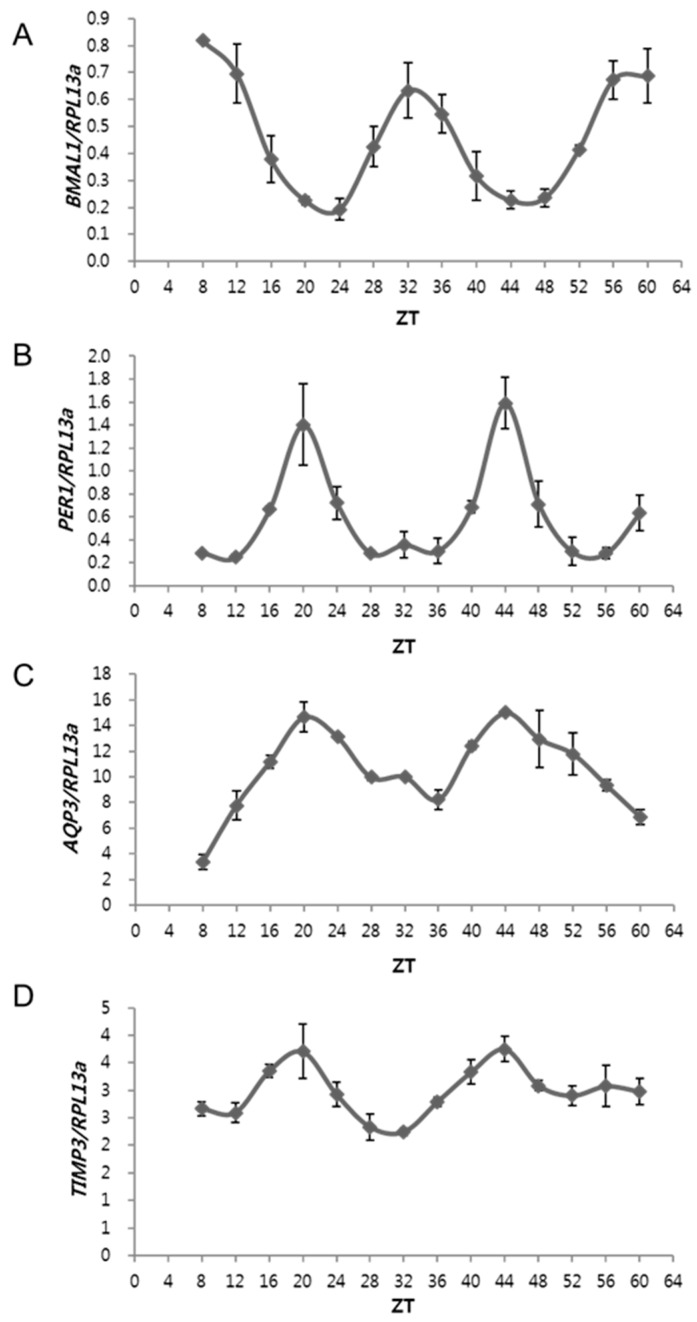
Circadian expression patterns of core clock and clock-controlled genes. Normal human epidermal keratinocytes (NHEKs) were synchronized in a serum-rich medium for 2 h and harvested every 4 h after replenishing with a basal medium. Gene expression patterns of core clock genes, *BMAL1* (**A**) and *PER1* (**B**), showed periodic oscillation, but were in antiphase to each other. Expressions of *AQP3*, (**C**), and *TIMP3,* (**D**), were oscillating periodically, similar to that of *PER1* but was antiphase to that of *BMAL1*. Y-axis represents mRNA abundance after being normalized to *RPL13a*; ZT indicates Zeitgeber Time (h).

We examined the circadian expression patterns of the core clock genes (BMAL1 and PER1) and epidermal CCGs (AQP3 and TIMP3) in human epidermal keratinocytes over time. Neonatal normal human epidermal keratinocytes (NHEKs) were synchronized using a serum-rich medium for 2 h (Zeitgeber time, ZT; ZT 0), and then harvested every 4 h for quantitative real-time PCR (qRT-PCR) analysis. BMAL1 showed the highest expression at ZT 8 and 32 (Figure 1A), but PER1 showed the highest expression at ZT 20 and 44 (Figure 1B), indicating that the circadian expressions of BMAL1 and PER1 were in antiphase in serum-synchronized human keratinocytes. AQP3, a known epidermal circadian regulatory and skin hydration-related gene, displayed a rhythmic expression similar to that of PER1, but was in antiphase to BMAL1 (Figure 1C). We recently identified TIMP3 as a novel clock-regulated gene based on RNA-seq analysis of CLOCK knockdown NHEKs [12]. TIMP3 displayed a rhythmic expression similar to those of PER1 and AQP3 (Figure 1D). The expression of core clock genes was previously reported in humans and rodents [13,14,15], demonstrating that PER1 had a peak expression at dawn in humans [13], and the circadian expression of BMAL1 and PER1 was in antiphase in human oral mucosa and skin samples [13]. Therefore, AQP3 and TIMP3, which show similar expression profiles to PER1, are also highly expressed during the daytime in the human skins and could be impacted by sunlight conditions. 

### 2.2. The Expression of AQP3 and TIMP3 Is Downregulated by UVB Irradiation and the Synthetic TIMP3-Peptides Inhibit UVB-Induced TNF-α Secretion

**Figure 2:**
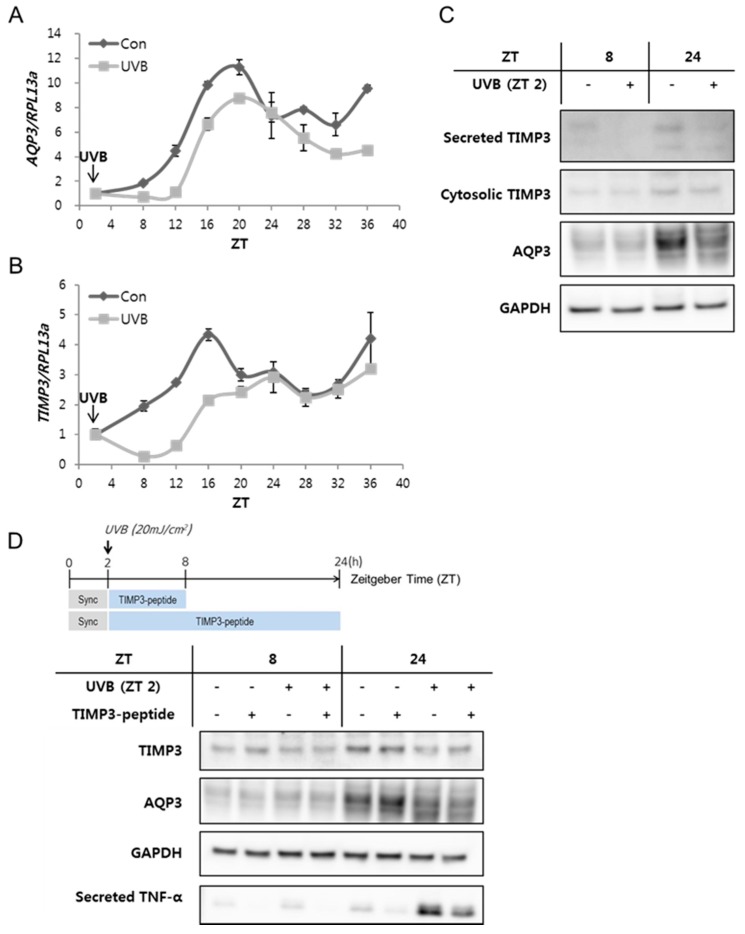
Expression of *AQP3* and *TIMP3* and the effect of synthetic metalloproteinase inhibitor 3 (TIMP3) peptides on tumor necrosis factor-α (TNF-α) secretion in ultraviolet B (UVB)-irradiation conditions. NHEKs were treated with 20 mJ/cm^2^ UVB at zeitgeber time (ZT) 2 and harvested every 4 h. The expression of *AQP3* (**A**) and *TIMP3* (**B**) was downregulated by UVB irradiation but recovered at ZT 24; (**C**) The secreted or cytosolic TIMP3 and aquaporin 3 (AQP3) were examined at ZT 8 and 24 after UVB irradiation. UVB irradiation reduced the protein levels of both TIMP3 and AQP3. Glyceraldehyde 3-phosphate dehydrogenase (GAPDH) was loaded as the control; (**D**) Synthetic TIMP3-peptides were used to treat the cells with or without UVB irradiation (Figure 3). TIMP3, AQP3, and the secreted TNF-α protein levels were analyzed using Western blotting at ZT 8 and 24. The synthetic TIMP3-peptides inhibited UVB-induced TNF-α secretion. GAPDH was loaded as control. Sync indicates synchronization.

The functional oscillation of clock genes can be disturbed by the environmental cue UVR [11]; thus, we introduced UVR conditions to NHEKs, to examine the circadian regulatory responses of *AQP3* and *TIMP3*. When NHEKs were exposed to UVB after a 2-h-synchronization period, the rhythmic expressions of *AQP3* and *TIMP3* transcripts were initially suppressed but recovered after 24 h post-treatment (Figure 2A,B), suggesting acute inhibitory effects of UVR on the rhythmic expressions of *AQP3* and *TIMP3*. The earlier reduction in the mRNA expression of each gene was followed by a decrease in the levels of secreted and cytosolic TIMP3 and AQP3 proteins at later time points (Figure 2C), suggesting a delayed rhythmic expression in protein levels compared to mRNA levels. A previous oligonucleotide microarray analysis study reported that the transcriptional response to UVB varied among genes depending on their function [10]. Some genes related to cell-adhesion and metabolism were downregulated, but the stress response, structural proteins, inflammatory response, mRNA splicing, proteasome-related proteins, and translation-related genes were upregulated by UV irradiation. The expression of core clock genes and of their downstream CCGs was proven to be disturbed by UVR [11], which in turn may be involved in UVR-induced gene regulation and cellular responses. 

TIMPs inhibit matrix metalloproteinases (MMPs) as well as disintegrin metalloproteinases, such as a disintegrin and metalloproteinase (ADAM) and a disintegrin and metalloproteinase with thrombospondin motifs (ADAMTS). Particularly, TIMP3, an inhibitor of ADAM17, can regulate cellular inflammation by negatively regulating the secretion of TNF-α, a pro-inflammatory cytokine, which is cleaved on the membrane by ADAM17 and released as an active soluble form [16]. Primary cytokines such as interleukin-1 (IL-1) and TNF-α secreted from activated immune cells and keratinocytes, evoke cytokine cascades related to inflammation. We examined if TIMP3 expression levels were related to the secreted TNF-α levels under UVB conditions. Upon exposure of NHEKs to UVB, the protein levels of TIMP3 and AQP3 were decreased, which was accompanied by the gradual increase of secreted TNF-α levels at ZT 8 and 24 (Figure 2D). Basal or UVB-induced TNF-α secretion at each ZT was reduced by the treatment with synthetic TIMP3-mimic peptides onto cells without changes in protein levels of TIMP3 and AQP3 (Figure 2D).

This result suggests that TIMP3 inversely regulates UVB-induced cellular responses such as the secretion of inflammatory factor TNF-α; therefore, TIMP3-mimic peptides or the materials to increase TIMP3 levels can be considered as potent effectors to inhibit UVR-induced cellular inflammation.

### 2.3. Camellia Sinensis Leaf Extract Can Recover the Downregulated TIMP3 Expression in UVB-Irradiated NHEKs

**Figure 3:**
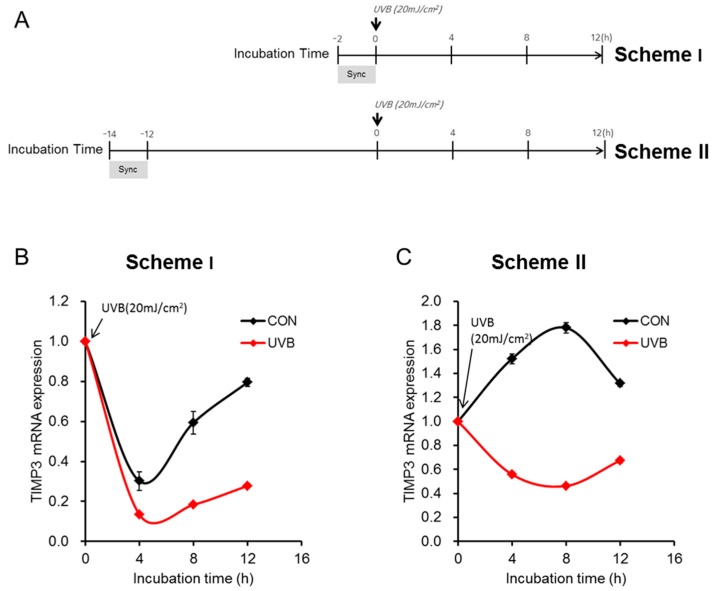
Schemes regarding the effects of UVB irradiation on TIMP3 expression depending on treatment. (**A**) Schemes for irradiation. After synchronization (Sync) of NHEKs by being incubated in a serum-rich medium (keratinocyte basal medium, KBM, supplemented with 50% horse serum) for 2 h, 20 mJ/cm^2^ of UVB was used for treatment immediately (Scheme I) or 12 h (Scheme II) post-synchronization; (**B**,**C**) NHEKs were irradiated following each scheme and harvested every 4 h. *TIMP3* expression was analyzed in each condition by qRT-PCR, showing differences between the control and UVB-treated groups following Scheme II; CON indicates control.

To identify the best condition to screen plant-derived materials that could increase TIMP3 expression under UVR conditions, we designed two schemes for UVB treatment: NHEKs were irradiated with UVB immediately or 12 h post-synchronization (Figure 3A, Scheme I and II, respectively), and the mRNA expression levels of *TIMP3* were examined every 4 h. In both schemes, *TIMP3* expression was downregulated within 4 h and slowly recovered 8 h after UVB irradiation (Figure 3B,C). However, the difference in mRNA expression between the control and UVB-irradiated conditions was much more dramatic in Scheme II (Figure 3C). Thus, we performed the screening assay using Scheme II to identify effective plant-derived materials to improve TIMP3 expression under UVB conditions.

**Figure 4:**
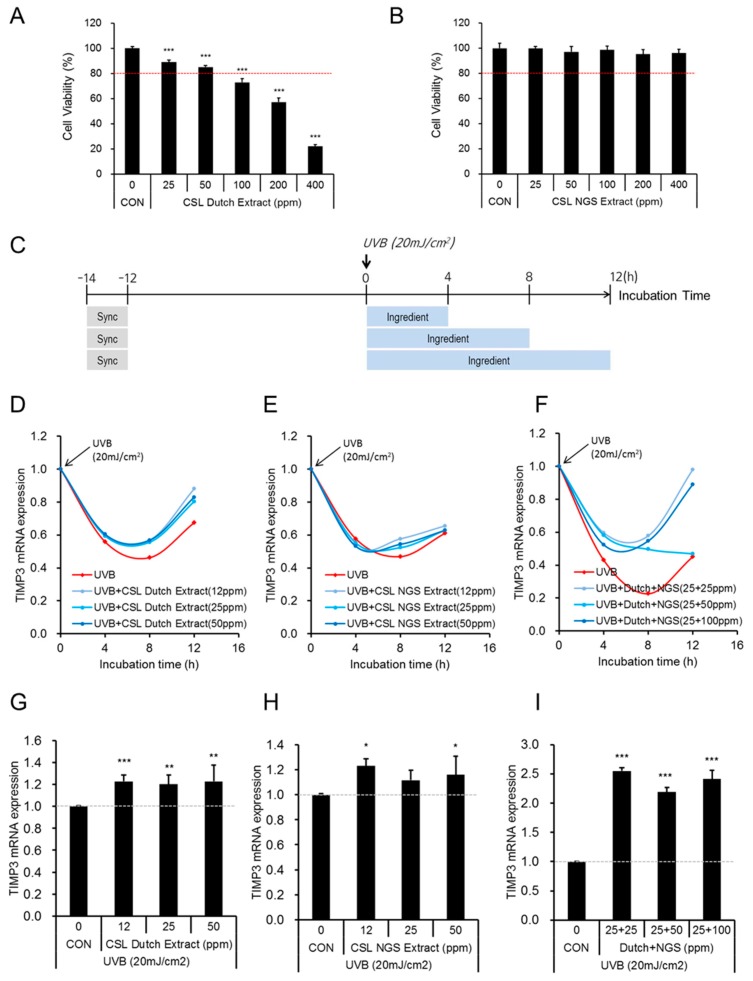
The effects of green tea extracts on down-regulated *TIMP3* mRNA expression in UVB-exposed human epidermal keratinocytes. For the cytotoxicity assay, NHEKs were incubated with 25–400 ppm of CSL Dutch extract (**A**), or 25–400 ppm of CSL new green solvent (NGS) extract (**B**), for 16 h. The data is presented as the mean ± S.D. of four replicates; *** indicates *p* < 0.001; CON indicates control; CSL indicates *Camellia sinensis* leaves; (**C**) A schematic flow chart of the experimental design to examine the effect of CSL extracts; Sync indicates synchronization; (**D**–**F**) After exposure to 20 mJ/cm^2^ of UVB, NHEKs were treated with CSL Dutch extracts (**D**), CSL NGS extracts (**E**), and Dutch + NGS extracts together (**F**), at the indicated doses and time periods. The expression of *TIMP3* mRNA in each treatment was quantified by quantitative real-time PCR (qRT-PCR); (**G**–**I**) Eight hours after the treatments with UVB and CSL extracts, the relative expression of *TIMP3* mRNA was compared in Dutch- (**G**), NGS- (**H**), and (Dutch + NGS)-treated (**I**) NHEKs. The data are presented as the mean ± S.D. of three replicates: *, *p* < 0.05; **, *p* < 0.01; and ***, *p* < 0.001.

Green tea (*Camellia sinensis*) leaves (designated as CSL) have been largely consumed by East Asian people for various purposes. Green tea extracts have benefits for stable maintenance of epigallocatechin gallate (EGCG), one of the major compounds of green tea [17]. These extracts are known for increasing procollagen synthesis and inhibiting MMP1 expression. We investigated if green tea extracts had the potential to recover the disrupted TIMP3 gene expression, due to the UVB irradiation. Two different types of green tea extracts were developed as cosmetic ingredients in our company: CSL Dutch extracts and CSL new green solvent (NGS) extracts. CSL Dutch extracts were slowly prepared by dropping the water at room temperature without stirring and CSL NGS extracts were prepared using the specific solvent (NGS; no EtOH included) with stirring. Based on the quantitative analyses of components using HPLC, CSL Dutch extracts contained higher amounts of amino acids compared to CSL extracts prepared using the water with stirring, but CSL NGS extracts contained higher amounts of catechins compared to general CSL Extracts prepared using an EtOH (70%)-extraction method with stirring. All extraction methods except the Dutch method included a stirring step to improve the extraction yield. Using two types of CSL extracts in the range of 25–400 ppm, we tested their cytotoxicities in NHEKs. Contrary to CSL Dutch extracts, which decreased cell viability significantly and gradually at high doses (100–400 ppm), CSL NGS extracts did not influence cell viability at the tested doses (Figure 4A,B). 

According to ISO 10993-5, substances eliciting a cell viability above 80% are considered to be non-cytotoxic. Therefore, we used doses of CSL Dutch (12, 25, and 50 ppm), resulting in over 80% of viability, or NGS (12, 25, 50, and 100 ppm) extracts separately or together, and examined the potency of each to recover the downregulated *TIMP3* expression in UVB-irradiated NHEKs. Using Scheme II irradiation conditions (Figure 3A), NHEKs were exposed to UVB at 12 h post-synchronization, and at the same time, the cells were treated with CSL Dutch extract, CSL NGS extract, or their combination for the indicated time periods (Figure 4C). *TIMP3* expression was maximally downregulated at 8 h post-irradiation, and the recovery effects of CSL extracts were also shown at the same time point (Figure 4D–F). Compared to the group treated with UVB alone, the group treated with CSL Dutch extracts for 8 h showed the recovery of the *TIMP3* expression by 22.8%, 20.3%, and 22.9%, at doses of 12, 25, and 50 ppm, respectively (Figure 4G). These recovery effects in *TIMP3* expression continued at 12 h post-irradiation. Compared to the control group, CSL NGS extracts also increased *TIMP3* expression by 23% and 15.9% at doses of 12 and 50 ppm at 8 h, respectively (Figure 4H), showing less effects than the CSL Dutch extracts. Compared to the group treated with UVB alone, when two types of CSL extracts (Dutch and NGS) were treated in a combination, *TIMP3* expression was dramatically upregulated, displaying 250% (25 + 5 ppm), 220% (25 + 50 ppm), and 240% (25 + 100 ppm) increases at 8 h post-irradiation (Figure 4I). Although the mode of action of the extracts in the regulation of *TIMP3* expression has not been pursued, these results suggest that the co-treatment of CSL Dutch and NGS extracts at low doses is a good strategy to recover the disrupted circadian expression of *TIMP3* under UVR conditions. Given that compared to general CSL extracts, CSL Dutch and NGS extracts are enriched with amino acids and catechins, respectively, the components of the other part introduced by the co-treatment may at least in part contribute to the enhanced effect on TIMP3 recovery under UVR conditions. Whether the recovery of TIMP3 expression by the co-treatment of CSL Dutch and NGS extracts is accompanied by a decrease in TNF-α secretion needs further investigation.

Through earlier and current studies, we identified *TIMP3* as a novel CLOCK-regulated gene and demonstrated its protective roles of inhibiting UVB-induced inflammation. Although we have focused on only UVR, there may be various conditions that disrupt TIMP3 expression, including hyperinflammation disorders such as psoriasis [18]. Considering the important roles of TIMP3 in skin homeostasis, the disrupted circadian *TIMP3* expression may be directly related to skin damages, including inflammation and aging; therefore, treatments using the different types of CSL extracts that were developed and their combination, will provide an alternative method to better care for the skin during the daytime, or even the night-time, to improve the circadian *TIMP3* expression.

## 3. Materials and Methods

### 3.1. Cell Culture, Synchronization, UVB Irradiation, and Treatments

NHEKs were purchased from Lonza (Clonetics Lonza, Walkersville, MD, USA) and maintained in keratinocyte growth medium (Keratinocyte Basal Medium (KBM) supplemented SingleQuots^TM^ Kit; Lonza, Basel, Switzerland) at 37 °C in a humidified atmosphere containing 5% CO_2_. NHEKs were synchronized as follows: NHEKs were grown in growth medium until the cells reached 70–80% confluency. The medium was then replaced with a serum-rich medium (KBM supplemented with 50% horse serum (Gibco)) and incubated for 2 h. The cells were collected for analysis of the mRNA and protein expressions at the indicated times. For UV irradiation, the cells synchronized in a serum-rich medium for 2 h were treated with 20 mJ/cm^2^ UVB, using BIO-SUN UV-H (VILBER LOURMAT, Collégien, France) in PBS solution, and the cells were then cultured back in fresh KBM. Immediately after the UVB irradiation, 10 ng/mL of human synthetic TIMP3-peptide (Abcam, Cambridge, UK) or *Camellia sinensis* leaf (CSL) extracts at different concentrations in KBM were used for the treatment. The CSL extracts used in this study were obtained from a new green tea tree species named *Jangwon No.3* [19] that was cultivated by AmorePacific Corporation. The cells were harvested at the indicated times for analysis of the mRNA and protein expressions of each gene. 

### 3.2. Viability Assay

For the cell viability assay, NHEK cells (7.0 × 10^4^ cells per well) were seeded onto a 24-well plate. After 24 h, each ingredient was used for treatment for an additional 16 h. The levels of cell viability were then estimated using the CCK-8 reagent (Dojindo Bio, Kumamoto, Japan). The results are graphically represented and show the relative cell viability normalized to the control (untreated) group (*n* = 4 per group).

### 3.3. Quantitative Real-Time PCR (qRT-PCR)

Total RNA was isolated using TRIzol reagent (Invitrogen, Carlsbad, CA, USA), and 2 μg of total RNA was used to synthesize cDNA using a reverse transcriptase kit (Invitrogen). Gene expression analyses were performed using the TaqMan universal master mix and TaqMan gene expression assays (Applied Biosystems, Foster City, CA, USA) in a 7500 fast real-time PCR system (Applied Biosystems) according to the manufacturer’s instruction. TaqMan gene expression assays consist of a pair of unlabeled PCR primers and a TaqMan probe with a dye label (FAM) on the 5′ end, and a minor groove binder (MGB) and non-fluorescent quencher (NFQ) on the 3′ end, and have a PCR efficiency of 100% (±10%), based on the manufacturer’s literature. The 60S ribosomal protein L13a (RPL13a) was used to normalize variations of cDNA quantities synthesized from different samples. A relative abundance in gene expression was determined using the 2^−ΔΔ*C*T^ method as previously described [20]. The gene name and its corresponding Taqman probe used in the Taqman gene expression assays are as follows; *RPL13a*: Hs04194366_g1, *BMAL1*: Hs00154147_m1, *PER1*: Hs00242988_m1, *AQP3*: Hs00185020_m1, *TIMP3*: Hs00165949_m1.

### 3.4. Western Blotting

For immunoblot analysis, cell lysates were separated on 4–12% gradient Bio-Tris gels and later transferred to nitrocellulose membranes (Invitrogen). The membranes were reacted with antibodies against TIMP3, TNF-α, AQP3 (Cell Signaling Technology, Beverly, MA, USA), or GAPDH (Santa Cruz Biotechnology, Santa Cruz, CA, USA). Anti-rabbit IgG was used as the secondary antibody (Santa Cruz Biotechnology). Blots were developed using the Western blotting luminol reagent (Santa Cruz Biotechnology).

### 3.5. Trichloroacetic Acid (TCA) Precipitation

To concentrate the secreted TIMP3 and TNF-α proteins from the cultured media of NHEKs at ZT 8 and 24 before or after UVB irradiation, 1 volume of 6.1 N trichloroacetic acid (TCA) (Sigma, St. Louis, MO, USA) was added to 10 volumes of cultured media. After an incubation on ice for 30 min, the cells were centrifuged at 13,000 rpm for 15 min. After removing the supernatant, the pellet was resuspended in the sample buffer, which was a mixture of sodium dodecyl sulphate-polyacrylamide gel electrophoresis (SDS-PAGE) staining buffer (Invitrogen) and 1 M Tris-HCl (pH 8.0), for immunoblot analysis. 

## 4. Conclusions

In summary, we showed that *TIMP3* mRNA oscillated periodically, similar to that of the core clock gene *PER1* and the epidermal circadian regulatory gene *AQP3*, but was in antiphase to the core clock gene *BMAL1*. This rhythmic expression was perturbed by UVB irradiation, which was followed by the increased secretion of TNF-α. TIMP3 synthetic peptides may play a protector role against UVB-induced cellular responses by inhibiting the secretion of TNF-α. As natural cosmetic ingredients, CSL Dutch extracts and CSL NGS extracts, partially recovered the disrupted circadian expression of *TIMP3*; however, the combinatorial treatment of both extracts showed the dramatic synergistic effects in UVB-irradiated keratinocytes. We proposed green tea extracts as natural products to improve circadian *TIMP3* expression in the skin and as a strategy for maximizing their effects.

## Abbreviations

UVR	Ultraviolet radiation
SCN	Suprachiasmatic nucleus
AQP3	Aquaporin 3
CCG	Clock-controlled gene
NHEK	Neonatal normal human epidermal keratinocytes
ZT	Zeitgeber Time
CSL	*Camellia sinensis* leaf
EGCG	Eigallocatechin gallate
NGS	New green solvent
KBM	Keratinocyte Basal Medium
qRT-PCR	Quantitative real-time PCR
TCA	Trichloroacetic acid

## References

[1] Ko C.H., Takahashi J.S. (2006). Molecular components of the mammalian circadian clock. Hum. Mol. Genet..

[2] Sporl F., Schellenberg K., Blatt T., Wenck H., Wittern K.P., Schrader A., Kramer A. (2011). A circadian clock in HaCaT keratinocytes. J. Investig. Dermatol..

[3] Dickmeis T., Weger B.D., Weger M. (2013). The circadian clock and glucocorticoids--interactions across many time scales. Mol. Cell Endocrinol..

[4] Yosipovitch G., Xiong G.L., Haus E., Sackett-Lundeen L., Ashkenazi I., Maibach H.I. (1998). Time-dependent variations of the skin barrier function in humans: Transepidermal water loss, stratum corneum hydration, skin surface pH, and skin temperature. J. Investig. Dermatol..

[5] Denda M., Tsuchiya T. (2000). Barrier recovery rate varies time-dependently in human skin. Br. J. Dermatol..

[6] Yosipovitch G., Sackett-Lundeen L., Goon A., Yiong Huak C., Leok Goh C., Haus E. (2004). Circadian and ultradian (12 h) variations of skin blood flow and barrier function in non-irritated and irritated skin-effect of topical corticosteroids. J. Investig. Dermatol..

[7] Sandu C., Dumas M., Malan A., Sambakhe D., Marteau C., Nizard C., Schnebert S., Perrier E., Challet E., Pevet P. (2012). Human skin keratinocytes, melanocytes, and fibroblasts contain distinct circadian clock machineries. Cell Mol. Life Sci..

[8] Matsunaga N., Itcho K., Hamamura K., Ikeda E., Ikeyama H., Furuichi Y., Watanabe M., Koyanagi S., Ohdo S. (2014). 24-hour rhythm of aquaporin-3 function in the epidermis is regulated by molecular clocks. J. Investig. Dermatol..

[9] Ando N., Nakamura Y., Aoki R., Ishimaru K., Ogawa H., Okumura K., Shibata S., Shimada S., Nakao A. (2015). Circadian Gene Clock Regulates Psoriasis-Like Skin Inflammation in Mice. J. Investig. Dermatol..

[10] Sesto A., Navarro M., Burslem F., Jorcano J.L. (2002). Analysis of the ultraviolet B response in primary human keratinocytes using oligonucleotide microarrays. Proc. Natl. Acad. Sci. USA.

[11] Kawara S., Mydlarski R., Mamelak A.J., Freed I., Wang B., Watanabe H., Shivji G., Tavadia S.K., Suzuki H., Bjarnason G.A. (2002). Low-dose ultraviolet B rays alter the mRNA expression of the circadian clock genes in cultured human keratinocytes. J. Investig. Dermatol..

[12] Park S., Kim K., Bae I.H., Lee S.H., Jung J., Lee T.R., Cho E.G. (2018). TIMP3 is a CLOCK-dependent diurnal gene that inhibits the expression of UVB-induced inflammatory cytokines in human keratinocytes. FASEB J..

[13] Bjarnason G.A., Jordan R.C., Wood P.A., Li Q., Lincoln D.W., Sothern R.B., Hrushesky W.J., Ben-David Y. (2001). Circadian expression of clock genes in human oral mucosa and skin: Association with specific cell-cycle phases. Am. J. Pathol..

[14] Oishi K., Sakamoto K., Okada T., Nagase T., Ishida N. (1998). Antiphase circadian expression between BMAL1 and period homologue mRNA in the suprachiasmatic nucleus and peripheral tissues of rats. Biochem. Biophys. Res. Commun..

[15] Lamont E.W., James F.O., Boivin D.B., Cermakian N. (2007). From circadian clock gene expression to pathologies. Sleep Med..

[16] Scheller J., Chalaris A., Garbers C., Rose-John S. (2011). ADAM17: A molecular switch to control inflammation and tissue regeneration. Trends Immunol..

[17] Osada K., Takahashi M., Hoshina S., Nakamura M., Nakamura S., Sugano M. (2001). Tea catechins inhibit cholesterol oxidation accompanying oxidation of low density lipoprotein in vitro. Comp. Biochem. Physiol. Part. C Toxicol. Pharmacol..

[18] Zibert J.R., Løvendorf M.B., Litman T., Olsen J., Kaczkowski B., Skov L. (2010). MicroRNAs and potential target interactions in psoriasis. J. Dermatol. Sci..

[19] Ji H.-G., Lee Y.-R., Lee M.-S., Hwang K.H., Kim E.-H., Park J.S., Hong Y.-S. (2017). Metabolic phenotyping of various tea (Camellia sinensis L.) cultivars and understanding of their intrinsic metabolism. Food Chem..

[20] Livak K.J., Schmittgen T.D. (2001). Analysis of Relative Gene Expression Data Using Real-Time Quantitative PCR and the 2^−ΔΔ*C*T^ Method. Methods.

